# Mental health of young Australians: dealing with a public health crisis

**DOI:** 10.5694/mja2.52047

**Published:** 2023-07-23

**Authors:** Patrick D McGorry, David Coghill, Michael Berk

**Affiliations:** ^1^ Centre for Youth Mental Health University of Melbourne Melbourne VIC; ^2^ Orygen Melbourne VIC; ^3^ University of Melbourne Melbourne VIC; ^4^ Murdoch Children's Research Institute Melbourne VIC; ^5^ Royal Children's Hospital Melbourne VIC; ^6^ Institute for Mental and Physical Health and Clinical Translation (IMPACT) Deakin University Barwon Health Geelong VIC; ^7^ Florey Institute of Neuroscience and Mental Health University of Melbourne Melbourne VIC

**Keywords:** Mental health services, Mental health policy

The mental health of young Australians is rapidly declining.^1^, ^2^ The evidence for this is increasingly solid and reflects a worldwide trend.^3^, ^4^, ^5^, ^6^, ^7^ This steady erosion of our collective mental wealth is not only a human tragedy but an economic one.^8^ Yet despite this worldwide megatrend, public and media discourse is muffled. Health and social care systems remain asymmetrically focused on physical illness and disability. Despite the erosive effect of mental illness, public pressure and, consequently, the political will for a response, in proportion to the scale and urgency of the crisis, are yet to materialise.

Mental illnesses are the chronic diseases of the young.^9^ Most adult mental disorders begin during the transition to adulthood.^10^ This pattern of onset, the extension of developmental transition to adulthood into the mid‐twenties, and the evidence that traditional primary care and specialist mental health services were simply not fit for purpose for young people led to innovative reform in youth mental health in Australia and, subsequently, other nations over the past two decades.^11^ However, although prescient, this has been nowhere near proportional to the scale of the problem.

Youth mental health is a moving target. Over the very period that youth mental health services focused on the 12–25‐year‐olds’ transition to adulthood began to be assembled, there has been an alarming rise in the incidence and prevalence of mental ill‐health in young people.^1^ The recent National Study of Mental Health and Wellbeing^1^ revealed that the prevalence of operationally defined mental disorders in 16–24‐year‐olds rose by 50%, from 26% in 2007 to 39% in 2021. The rise in young women was more marked than in young men, with rates reaching 48%. The HILDA survey of 17 000 households/people confirmed a long term decline in the mental health of this age group.^2^ This survey also captured the additional impact of the coronavirus disease 2019 (COVID‐19) pandemic and its mitigation strategies.^2^ Similar alarming trends had been identified in many other high income countries well before the COVID‐19 pandemic.^3^, ^6^, ^7^, ^12^ In 2021, in an advisory to the United States President and Congress, the US Surgeon General, responding to similar trends in the US, described the situation as a “youth mental health crisis”.^13^

The consequences of this rising tide of mental ill‐health are profound. We invest heavily to bring young people to the threshold of productive adult life. This nurturing of human potential represents the creation of “mental wealth”.^8^ Yet this wealth is being squandered. Mental ill‐health weakens psychosocial maturation, relationships, educational attainment, workplace culture, and productivity.^14^ Suicide is the leading cause of death in young people^15^ and may be rising again post‐pandemic.^16^ Severe mental illness additionally reduces life expectancy by up to 20 years through a combination of premature physical illness and suicide.^17^ Mental illness due to its timing in the life cycle and the receding tide of infectious and many physical disorders such as cardiovascular disorders is now the number one cause of disability and of chronic disease in Australia.^18^ Early intervention for potentially disabling illnesses safeguards mental wealth, notably with psychosis (where the return on investment can be as high as 17:1),^19^ but also anxiety, depression, and attention deficit/hyperactivity disorder (ADHD).^20^

## Current responses

Australia has been ahead of the curve in initiating reform, implemented by successive federal governments with the design and scaling up of headspace centres within over 150 communities across all states and territories of Australia. Headspace is a “soft entry” enhanced primary care service system for 12–25‐year‐olds who present with a range of health and social issues, predominantly mental health‐related.^11^ As a one‐stop‐shop, it offers horizontal integration of health and social services, with codesign from young people to create and safeguard a youth‐friendly culture of care. It has inspired widespread global reform in youth mental health primary care.

Despite some criticism,^21^ a series of independent evaluations have demonstrated that headspace improves access, safety, acceptability and outcomes, and is cost‐effective for the earlier stage and mild to moderate levels of need for which it was designed.^22^, ^23^, ^24^ Nevertheless, headspace is well overdue for redesign and refinancing. The rising tide of new cases is now overwhelming the capacity to provide timely access and the funding model has been exposed as no longer adequate to recruit and retain a workforce,^25^ given that other more lucrative options exist.

A comprehensive youth mental health system involves much more than entry level primary care. Young people with more severe, complex or persistent conditions need more expert, sustained and intensive care. Yet this next level of secondary care is largely absent, resulting in a large cohort of young people described as the “missing middle”.^26^ This second tier of care would allow primary care to effectively focus on those with mild to moderate mental health needs and strengthen engagement outcomes. The Youth Enhanced Services (YES) initiative funded from 2016 by the federal government is a weak and piecemeal response commissioned by Primary Health Networks (PHNs) with little regard to evidence or integration with headspace centres or state services. With the exception of Victoria, which is expanding and restructuring its specialist mental health system to create a subsystem of care for 12–25‐year‐olds to vertically integrate with headspace, state public systems persist with an outdated child and adolescent mental health and adult mental health service model split with a boundary at 18 years, which, despite the best efforts of dedicated staff, is obsolete and fundamentally flawed.^27^, ^28^, ^29^, ^30^

Although mental illness is the largest cause of disability,^18^ it is poorly served by the National Disability Insurance Scheme (NDIS). Early intervention and recovery — essential principles of youth mental health — are in direct conflict with the current NDIS model,^31^ which therefore makes any return on investment impossible. The “catch 22” that requires any disability to be not merely severe but end‐stage and permanent means that young people who might ultimately meet that definition are deprived of the very support they need to avoid such devastating outcomes. In this way the scheme is self‐defeating. Young people have the greatest capacity to benefit, yet most cannot even get to the starting blocks. Only 3% of the mental health packages awarded by the scheme are offered to young people aged 12–25 years.^32^ Furthermore, for those fortunate enough to board the NDIS lifeboat, first order needs, such as secure accommodation, meaningful social and employment roles, and quality mental health care, are often not within the envelope of coverage. This is inefficient and wasteful. However, since the average NDIS package for mental illness is vastly more generous than what the health system spends on the mental health care of people with severe mental illness, if this were combined and spent on their real clinical needs, the outcomes could be transformed.

This highlights a long‐standing public policy weakness: health and social care lack integration, due to a long‐standing artificial split created between “clinical” and “psychosocial” care. Both streams should be delivered through a single holistically configured service. Health policy in recent years has further accelerated fragmentation. Since 2016, commissioning of aspects of federally funded health care, especially mental health care, has been outsourced to essentially privatised PHNs, which employ market‐inspired competitive tendering processes which are not fit for purpose. This commissioning model inhibits integrative care, instead spawning a bewildering array of competing, siloed and often rotating and transient non‐government organisations which deliver “salami slices” of health and social care.^14^, ^33^ This ecosystem is diffuse, piecemeal, poorly integrated and regulated, typically non‐evidence‐based, confusing to the public, and, ultimately, ineffective. Money is wasted and the unique opportunity for the return on investment that improved mental health offers is missed. This approach and especially the outsourced PHN model of commissioning needs a fundamental rethink.

## Solutions

How should we respond to this alarming and unacceptable situation? There are four avenues for reform and investment.

The first involves prevention. Given the alarming increase in mental disorders, particularly in young women,^1^ we need to rapidly understand which of the megatrends in global society are singly or jointly responsible. The answers are likely to involve a blend of socio‐economic and generational changes, rising adversity and inequality, and unforeseen consequences of technological advances.^34^ The US Surgeon General,^13^ drawing in part on the research and advocacy of Jean Twenge,^5^ has identified social media as a key megatrend undermining the mental health of young people. Other experts have pointed out that it is likely to be merely one influence and not the whole story behind the youth mental health crisis.^34^ There is an urgent need to gain clarity on the malleable risk factors and megatrends driving the decline in mental health.

The second focus is early intervention. Integrated primary youth mental health care services are a focal point for early intervention,^11^ which aim to speed access within the mild to moderate early stages of mental ill‐health. Not only can milder, less persistent illnesses be successfully treated, but treatment delays for more severe and disabling illnesses can also be reduced and intervention for potentially severe mental illness made possible during a more treatment‐responsive stage. Primary care generally needs reimagining and a new financial model. The surge in need, workforce shortages and the collapse of bulk‐billing has created a perfect storm both for GPs and headspace centres. Rapid, stigma‐free access is no longer possible. For the headspace system, a return to nationally coherent commissioning, a new financial model less reliant on fee‐for‐service bulk‐billing, and financial parity with the new adult Head to Health community mental health model are all needed.

Third, rather than an automatic second batch of Better Access sessions,^35^ most young people with more severe and persistent illness need expert, multidisciplinary team‐based care to recover. This requires a more specialised tier of care, a back‐up system for primary care providers for young people, so far only available in a small number of “oasis” zones. The Early Psychosis Youth Services (EPYS) program, linked to primary care, exists in only eight regions of Australia. This cost‐effective model^36^ should expand its diagnostic coverage to other complex disorders and be progressively assembled in all regions as a vital bridge for the “missing middle” between primary care and state‐funded tertiary care. This would enable the YES program to be wound up and the resources channelled to help support an evidence‐based model. Expanded professional workforces need to be tied to team‐based multidisciplinary care rather than fragmented fee‐for‐service solo private practice models. The federal government already possesses solid blueprints for such secondary care models that can be further refined in the lead up to mid‐year economic and fiscal outlook and the 2024 budget.

Finally, the official federal government review of the NDIS now underway^37^ is considering how the mental illness component of the NDIS should be redesigned both at tier 1 and tier 2 levels.^37^ Funding from either or both tiers could be channelled to cofund and greatly augment the fully integrated care model described above which enables health and social care to be horizontally integrated under one governance structure and within the same service. Young people in the early stages of potentially disabling mental illnesses, including treatable neurodevelopmental disorders such as ADHD, should be prioritised and no longer excluded. This integrated model should also be considered to augment and revive the adult Head to Health centre program.

These measures would transform outcomes in youth mental health and create return on investment such that federal budget repair and the wellbeing of society would be strengthened.

## Open access

Open access publishing facilitated by The University of Melbourne, as part of the Wiley ‐ The University of Melbourne agreement via the Council of Australian University Librarians.

## Competing interests

Patrick McGorry is a founding director of headspace and executive director of Orygen, Australia's National Centre of Excellence in Youth Mental Health. Orygen is the lead agency for five headspace centres across northwest Melbourne.

## Provenance

Not commissioned; externally peer reviewed.
